# The Muscarinic Acetylcholine M_2_ Receptor-Induced Nitration of p190A by eNOS Increases RhoA Activity in Cardiac Myocytes

**DOI:** 10.3390/cells12202432

**Published:** 2023-10-11

**Authors:** Magdolna K. Levay, Lena Throm, Nabil Bahrami, Thomas Wieland

**Affiliations:** 1 Experimental Pharmacology Mannheim (EPM), European Center for Angioscience (ECAS), Medical Faculty Mannheim, Heidelberg University, 68167 Mannheim, Germany; magdolna.levay@medma.uni-heidelberg.de (M.K.L.); lena.throm@t-online.de (L.T.); nabil.bahrami@medma.uni-heidelberg.de (N.B.); 2 German Center for Cardiovascular Research (DZHK), Partner Site Heidelberg/Mannheim, 68167 Mannheim, Germany

**Keywords:** muscarinic acetylcholine receptor (MR), endothelial nitric oxide synthase (eNOS), caveolin-3 (Cav3), p190RhoGAP, p190A, p190B, regulator of G protein signaling 3 (RGS3), Rac1, RhoA

## Abstract

p190RhoGAP, which exists in two paralogs, p190RhoGAP-A (p190A) and p190RhoGAP-B (p190B), is a GTPase activating protein (GAP) contributing to the regulation of the cellular activity of RhoGTPases. Recent data showed that M_2_ muscarinic acetylcholine receptor (M_2_R) stimulation in neonatal rat cardiac myocytes (NRCM) induces the binding of p190RhoGAP to the long isoform of the regulator of G protein signaling 3 (RGS3L). This complex formation alters the substrate preference of p190RhoGAP from RhoA to Rac1. By analyzing carbachol-stimulated GAP activity, we show herein that p190A, but not p190B, alters its substrate preference in NRCM. Based on data that the RhoGAP activity of p190A in endothelial cells is diminished upon nitration by endothelial nitric oxide synthase (eNOS)-derived peroxynitrite, we studied whether carbachol-induced NO/peroxynitrite formation contributes to the carbachol-induced RhoA activation in NRCM. Interestingly, the carbachol-induced RhoA activation in NRCM was suppressed by the eNOS-preferring inhibitor L-NIO as well as the non-selective NOS inhibitor L-NAME. Using L-NIO, we firstly verified the carbachol-induced NO production concurrent with eNOS activation and, secondly, the carbachol-induced nitration of p190A in NRCM. By co-immunoprecipitation, the carbachol-induced complex formation of eNOS, p190A, RGS3L and caveolin-3 was detected. We thus conclude that the NO production by M_2_R-induced eNOS activation in caveolae in NRCM is required for the nitration of p190A, leading to the binding to RGS3L and the change in substrate preference from RhoA to Rac1. In line with this interpretation, the disruption of caveolae in NRCM by methyl-β-cyclodextrin suppressed carbachol-induced RhoA activation in NRCM to a similar extent as the inhibition of NO production.

## 1. Introduction

Nitric oxide synthases (NOS) regulate the transmembrane signaling of different G-protein-coupled receptors and have a wide spectrum of physiological roles. Endothelial nitric oxide synthase (eNOS), the isoform that was originally characterized in endothelial cells, is present in cardiac myocytes, the vascular and endocardial endothelium and the sympathetic and parasympathetic autonomic nerves and plays a critical role in regulating and maintaining a healthy cardiovascular system [1,2]. Nitric oxide (NO)—a highly reactive autocrine and paracrine signaling molecule with complex and controversial effects, produced by eNOS in the cardiac myocytes—is generally considered to be cardioprotective [3,4]. Polymorphisms in the eNOS gene, altered eNOS function and eNOS uncoupling contribute to different cardiovascular diseases, including high blood pressure and heart failure [1,5]. Thus, eNOS-deficient mice develop hypertension and progressive left ventricular hypertrophy associated with alteration of calcium handling protein expression [6,7]. 

Interestingly, eNOS is targeted to caveolae in endothelial cells as well as in cardiac myocytes [7,8]. Caveolae are specialized microdomains of the plasma membrane containing caveolins, which are key structural components and serve as a scaffold for signaling proteins [9]. Many of these signaling molecules within caveolae are involved in cardiac protection [10]. One of them is the M_2_ muscarinic acetylcholine receptor (M_2_R)—the quantitatively dominant isoform in cardiac tissues—which translocates into caveolae upon agonist binding, which leads to the activation of eNOS in cardiac myocytes [11,12]. The M_2_R-induced signaling is under the control of various regulatory proteins. For example, we recently demonstrated that the long isoform of the regulator of G protein signaling 3 (RGS3L) redirects the G_i_-mediated Rac1 activation into G_i_-mediated RhoA activation after M_2_R activation by complex formation with the GTPase-activating protein (GAP) p190RhoGAP and changing its substrate specificity. This switch leads to the reduced production of reactive oxygen species in cardiomyocytes and an increased Rho-dependent kinase-mediated inotropic response, as observed in an ex vivo experimental setting [13]. 

Two closely related and ubiquitously detected paralogs of p190RhoGAP, p190A (ARHGAP35) and p190B (ARHGAP5), exist, which have partially overlapping but also distinct functions [14]. For example, in endothelial cells [15,16], Siddiqui et al. showed that p190A, but not p190B, is a target downstream of eNOS-mediated NO production and subsequent peroxynitrite formation. The eNOS-derived peroxynitrite nitrated p190A at Tyr1105, which resulted in impaired GAP activity towards RhoA. The increased activity of RhoA led to adherence junction disassembly and an increase in endothelial permeability [16]. 

Based on data showing that M_2_Rs induce eNOS activation and NO production in the caveolae of cardiac myocytes [11], we hypothesized that the subsequent local peroxynitrite formation in proximity to caveolar M_2_Rs might mediate the observed switch in the GAP activity of p190A by nitration, contributing to the reported enhanced RhoA and decreased Rac1 activity in cardiac myocytes [13,17]. We therefore studied, in the model of neonatal rat cardiomyocytes (NRCM), whether the stimulation of M_2_R by carbachol induces eNOS activation and the nitration of p190A dependent on the presence of RGS3L. We also investigated the role of the cardiac caveolar scaffold caveolin-3 (Cav3) as a known complex partner of eNOS [11]. We finally analyzed whether the anticipated reduced GAP activity of p190A towards RhoA causes a carbachol-induced increase in RhoA activity sensitive to eNOS inhibition and treatments known to induce caveolae disruption. 

## 2. Materials and Methods

### 2.1. Antibodies, Reagents, and Inhibitors

We used the following primary antibodies: mouse-anti-RhoA (26C4, Santa Cruz, Heidelberg, Germany, sc-418), mouse-anti-Rac1 (BD Transd. Laboratories, Heidelberg, Germany, 610650), mouse-anti-p190RhoGAP (p190A) (BD Transd. Laboratories, 610149), mouse-anti-p190B (BD Transd. Laboratories, 611612), mouse-anti-RGS3 (CC-Q7, Santa Cruz, sc-100762), mouse-anti-caveolin-3 (BD Transd. Laboratories, 610421), mouse immunoglobulin G (IgG) (Santa Cruz sc-2025), rabbit-anti-caveolin-3 (H-100, Santa Cruz, sc-28828), mouse-anti-eNOS (BD Transd. Laboratories, 610297), rabbit-anti-phospho-1177Ser-eNOS (Cell Signaling Technology, Frankfurt, Germany, #9571), rabbit-anti-Nitrotyrosine (Millipore, Darmstadt, Germany, 06-284). The corresponding horseradish peroxidase-conjugated secondary antibodies were from Sigma-Aldrich (Munich, Germany, A-9044, A-9169). In this study, the following reagents and inhibitors were used: carbamoylcholine chloride (carbachol, Sigma-Aldrich, Munich, Germany, C4382), 5-bromo-2′-deoxyuridine (5-BrdU) (Sigma-Aldrich), methyl-β-cyclodextrin (MβCD, Sigma-Aldrich, M7439), (2S)-2-amino-5-(1-aminoethylideneamino)pentanoic acid; dihydrochloride (L-NIO dihydrochloride, Santa Cruz, sc-361229), Nω-nitro-L-arginin-methylester-hydrochloride (L-NAME, Sigma-Aldrich, 72760).

### 2.2. Isolation and Culture of Neonatal Rat Cardiac Myocytes 

Neonatal rat cardiac myocytes (NRCM) were isolated from the ventricles of 1 to 3 days old neonatal Wistar rats, as described previously [13]. The care and experimental use of all animals in this study were in accordance with the ARRIVE guidelines and approved by the local ethics committee (Regierungspraesidium Karlsruhe). 

Briefly, ventricles were separated from the atria and were minced and subjected to serial digestion in a mixture of collagenase (0.5 mg/mL collagenase type II, Cell Systems) and pancreatin (0.6 mg/mL, Sigma-Aldrich) to release single cells. The obtained cell suspension was placed on top of a Percoll gradient (Cytiva, Uppsala, Sweden) to separate cardiac myocytes from other cell types. The cardiac myocyte fraction was seeded on collagen-I-coated plates and cultured in DMEM supplemented with 10% (*w*/*v*) fetal bovine serum (FBS), 2 mM L-glutamine, 100 units/mL penicillin, and 100 μg/mL streptomycin in a humidified atmosphere of 5% CO_2_ at 37 °C. Then, 0.1 mM 5-BrdU was used to prevent the overgrowth of other, non-cardiac myocyte cell types. The cells were used for experiments between 3 and 5 days after isolation. Serum-reduced conditions (DMEM supplemented with 0.5% FBS) were used for 48 h when indicated.

### 2.3. Immunoblot Analysis

For immunoblot analysis, the protein samples were separated by SDS-PAGE using 8–15% denaturing acrylamide gels and transferred onto nitrocellulose membranes. Membranes were blocked with Roti-Block (Carl Roth, Karlsruhe, Germany) for 1 h at room temperature and incubated with specific primary antibodies (see above) overnight at 4 °C and according to the manufacturer’s recommendations. After incubation with appropriate secondary antibodies for 1 h, proteins were visualized by enhanced chemiluminescence using an imaging system (Alpha Innotech, Wiesbaden, Germany). The Image J software, v1.54f, was used for the analysis of the blots.

### 2.4. RhoGTPase Activation Assay

The cellular RhoA-GTP levels were measured with a pull-down assay using a GST fusion protein containing the Rho-binding domain of rhotekin (GST-RBD). GST-RBD was expressed and purified from *E. coli.* NRCM were transduced with a control EGFP-adenovirus or an adenovirus encoding the GAP-deficient RGS3L mutant RGS3L-N460A and pretreated with the indicated inhibitors. The generation of the adenoviruses was described previously [13,17]. After activation of the NRCM with carbachol (1 mM, 5 min), cells were lysed in ice-cold GST-Fish buffer [17] and pelleted by centrifugation (12,000 rpm, 3 min at 4 °C). The GTP-bound RhoA contained in the supernatant was incubated for 1 h at 4 °C with GST-RBD bound to glutathione–sepharose beads. The beads were pelleted by centrifugation. After twice washing of the beads, bound proteins were eluted with sample buffer and separated by SDS-PAGE. The amount of activated RhoA was determined by immunoblot analysis as described above. Total RhoA from lysates was used as a loading control.

### 2.5. Immunoprecipitation

NRCM were transduced with RGS3L-N460A adenovirus for 24 h and stimulated with 1 mM carbachol or solvent for 5 min at 37 °C. Co-immunoprecipitation was performed as described previously [13]. Briefly, cells were lysed with immunoprecipitation buffer (50 mM Tris-HCl, pH 7.4, 2 mM EDTA, 150 mM NaCl, 0.1% SDS, 1% Nonidet P-40, 10 mM NaF) containing 1 mM sodium orthovanadate, 1 mM Pefablock, 10 µg/mL aprotinin, 10 µg/mL leupeptin. After centrifugation, the cleared lysates were incubated with the indicated antibodies (2 μg) under agitation for 1 h at 4 °C. After the addition of 40 μL 1:1 (*v*/*v*) protein-A–sepharose beads (Amersham Biosciences, Freiburg, Germany), the mixture was gently shaken for an additional 3–4 h at 4 °C. Beads were washed three times with immunoprecipitation buffer and eluted in SDS-containing buffer for 5 min at 95 °C. After SDS-PAGE and transfer to nitrocellulose membranes, immunoprecipitated proteins were detected by Western blot analysis using the indicated antibodies, according to standard protocols. Final detection was done with an ECL system (Amersham); the band intensity was quantified with the ImageJ software.

### 2.6. Fluorescence Determination of Nitric Oxide Production in NRCM

NO production in NRCM was measured using 4-amino-5-methylamino-29,79-difluorofluorescein (DAF-FM, Molecular Probes, Eugene, OR, USA) following the manufacturer’s instructions. Briefly, NRCM were cultured on 96-well black plates (Sarstedt, Nümbrecht, Germany). Thereafter, the cells were stimulated with 1 mM carbachol or solvent for 5 min at 37 °C. Cells were incubated with 10 µM DAF-FM for 2 h at 37 °C. Supernatants were then removed, and cardiomyocytes were washed with DAF-FM-free buffer. DAF-FM fluorescence was measured using the Envision 2102 Multilabel Reader with a set of FITC filters (excitation 485 ± 10 nM and emission at 535 ± 20 nM, PerkinElmer, Rodgau, Germany), respectively. Results are presented as the percentage of control.

### 2.7. Measurement of the Substrate Specificity of p190A and p190B towards Rac1 and RhoA

Measurement of the substrate preference of p190A and p190B was performed by a pulldown assay using constitutively active RhoA (RhoAQ63L) or Rac1 (Rac1Q61L), as described before [13,18]. Constitutively active monomeric GTPases bind to their respective GAPs, but as GTP hydrolysis is blunted by the mutation of the monomeric GTPase, the GAP/GTPase complex is stable. Therefore, the binding of p190A or p190B to RhoAQ63L-GST- or RacQ61L-GST-coated sepharose beads was analyzed by subsequent immunoblotting using isoform-specific antibodies.

### 2.8. Statistical Analysis

Data were expressed as mean + SD calculated from n assays and analyzed using the GraphPad Prism software (GraphPad Software, Version 6, La Jolla, CA, USA). Student’s *t*-test/nonparametric test or ANOVA plus an appropriate posttest for multiple comparisons were used for statistical analysis. A *p* value  <  0.05 was considered statistically significant.

## 3. Results and Discussion

**Figure 1:**
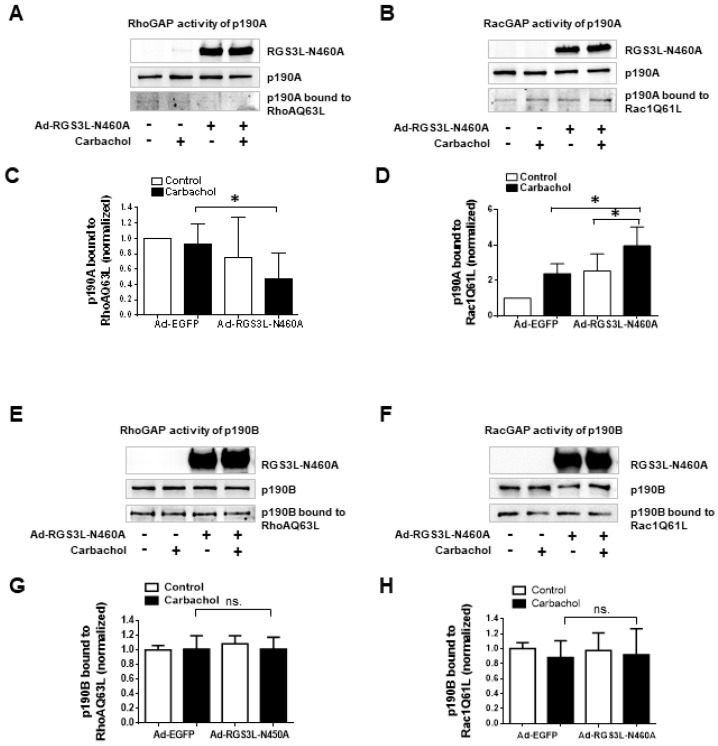
Measurement of the RhoGAP and RacGAP activity of p190A and p190B in NRCM. Here, 48 h after transduction with Ad-RGS3L-N460A or Ad-EGFP, NRCM were incubated with or without 1 mM carbachol for 5 min. Functionally active p190A (**A**–**D**) or p190B (**E**–**H**) was precipitated from the cell lysates with constitutive active RhoAQ63L-GST or constitutive active Rac1Q61L-GST-coated beads. Anti-p190A antibody or an anti-p190B-antibody were used to detect active p190A and p190B in the precipitates. The total amount of p190A or p190B in the lysates served as a loading control. Relative RhoGAP/RacGAP activity was calculated from the amount of p190A and p190B bound to RhoAQ63L- or Rac1Q61L-coated beads normalized to the total amount of p190A and p190B in the cell lysates, respectively. Representative experiments for p190A (**A**,**B**) and p190B (**E**,**F**) as well as quantification of the p190A activity (**C**,**D**) and p190B activity (**G**,**H**) are shown. The mean of the control value was set to 1.0 in each individual experiment. The other values are shown as mean ± SD of *n* = 9 (**C**), *n* = 7 (**D**) or *n* = 6 (**G**), *n* = 7 (**H**) independent experiments. One-way ANOVA with Tukey’s multiple comparison was performed for these values, * *p* < 0.05.

GTPase-activating proteins, e.g., p190RhoGAP, which is known to have both RhoGAP and RacGAP activity, are important regulators of the Rho family GTPases. Moreover, p190RhoGAP is able to switch its substrate preference and thus alter the Rac1–RhoA activity balance in cells [13,19]. We have recently shown that in cardiac myocytes, such as NRCM, the increased expression of RGS3L redirects the M_2_R-mediated Rac1 activation to the activation of RhoA. Similar effects were obtained by increasing the endogenous RGS3L expression by stimulation of NRCM with fibroblast growth factor 2 for 24 h or adenoviral overexpression of the mutant RGS3L-N460A [13]. As an underlying mechanism, we identified the carbachol-induced complex formation of RGS3L with p190RhoGAP, which increased the GAP activity towards Rac1 and reduced the activity towards RhoA [13]. As both closely related paralogs of p190RhoGAP, p190A and p190B, are expressed in NRCM, we first studied whether only one or both paralogs were regulated in their substrate preference upon carbachol stimulation. We transduced NRCM with control EGFP-adenovirus or an adenovirus encoding the GAP-deficient RGS3L mutant RGS3L-N460A, which mediates, in M_2_R-expressing cells, prolonged carbachol-induced RhoA activation compared to wild-type RGS3L in M_2_R-expressing cells [17]. We then analyzed the binding of p190A and p190B to permanently active Rac1Q61L or RhoAQ63L using isoform-specific antibodies. In accordance with the reported data [13], carbachol increased the binding of p190A to Rac1Q61L (Figure 1) in cells expressing high levels of RGS3L, whereas the binding to RhoAQ63L was decreased in contrast. The binding of p190B to Rac1Q61L and RhoAQ63L remained unchanged by the high expression levels of RGS3L, as well as the stimulation of NRCM with carbachol, indicating that only the substrate preference of p190A can be switched depending on the interaction with RGS3L.

### 3.1. The Substrate Preference of p190A towards RhoGTPases, as Well as the Complex Formation with RGS3L, Depends on Nitration via eNOS-Derived Peroxynitrite Formation

**Figure 2:**
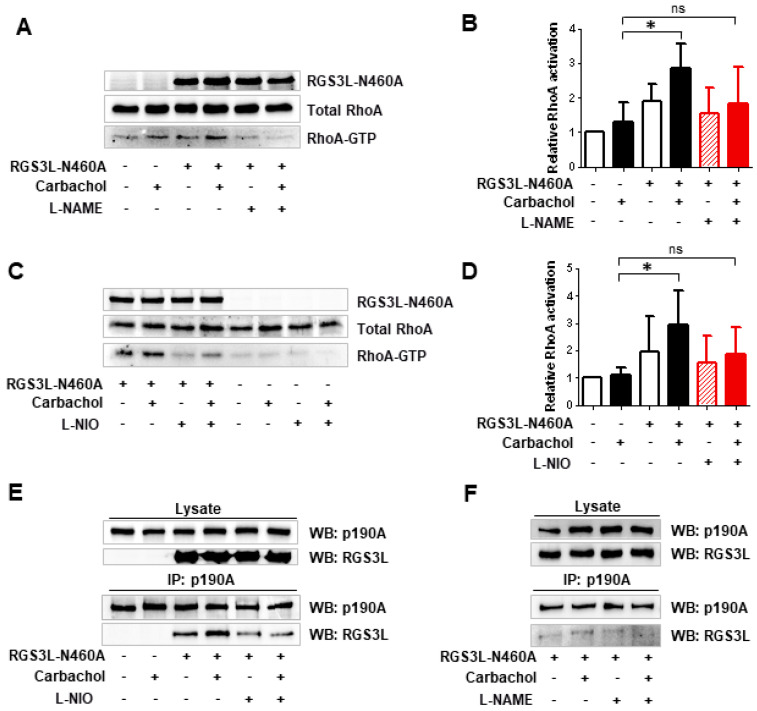
Effect of the inhibition of eNOS on the carbachol-induced RhoA activation and RGS3L/p190A complex formation in NRCM. NRCM were transduced with Ad-RGS3L-N460A or Ad-EGFP for 48 h. Before stimulation with or without 1 mM carbachol for 5 min, cells were treated without and with 10 µM L-NAME (**A**,**B**) for 10 min or without and with 10 µM L-NIO (**C**,**D**) for 15 min. Level of RhoA-GTP was measured by the RhoA activation assay. Quantification of RhoA activity (RhoA-GTP/total RhoA) (**B**,**D**) as well as representative experiments (**A**,**C**) are shown. The mean RhoA-GTP level detected in unstimulated cells was set to 1.0. Values are mean ± SD, *n* = 8 (**B**), *n* = 7 (**D**) * *p* < 0.05; one-way ANOVA with Tukey’s multiple comparison was performed. Visualization of the effect of eNOS inhibition on the interaction of p190RhoGAP with RGS3L in NRCM by co-immunoprecipitation (**E**,**F**). NRCM were transduced with Ad-EGFP or Ad-RGS3L-N460A, treated with 10 µM L-NIO (**E**), 10 µM L-NAME (**F**) or solvent for 15 min, and stimulated without and with carbachol for 5 min. Immunoprecipitation was performed using the anti-p190A antibody. Co-precipitated RGS3L was visualized by immunoblotting. The total amount of p190A in the lysates served as a loading control.

In endothelial cells, peroxynitrite formation from increased NO production upon eNOS activation nitrated p190A but not its paralog p190B. The fact that the nitration of p190A occurred on Tyr1105 was proven by using a p190A-Y1105A mutant. As a consequence of Tyr1105 nitration, the GAP activity of p190A towards RhoA was reduced, leading to increased RhoA activation [16]. Based on these data, we hypothesized that the decrease in RhoGAP activity observed by the carbachol-induced complex formation with RGS3L in NRCM might similarly require the nitration of p190A and thus depends on eNOS-derived NO formation. To prove the possible involvement of eNOS, we first treated NRCM with the broad-spectrum NOS inhibitor L-NAME or the more eNOS-specific inhibitor L-NIO (Figure 2). As shown before [13,17,20], carbachol-induced RhoA activation was detected in cells with high expression levels of RGS3L. This carbachol-induced RhoA activation was largely suppressed by treatment with L-NAME (Figure 2A,B) or L-NIO (Figure 2C,D). We therefore studied the influence of L-NIO on the carbachol-induced complex formation of RGS3L with p190A. As shown in Figure 2E,F, the carbachol-dependent complex formation was reduced when NRCM were treated with L-NIO and L-NAME, respectively.

**Figure 3:**
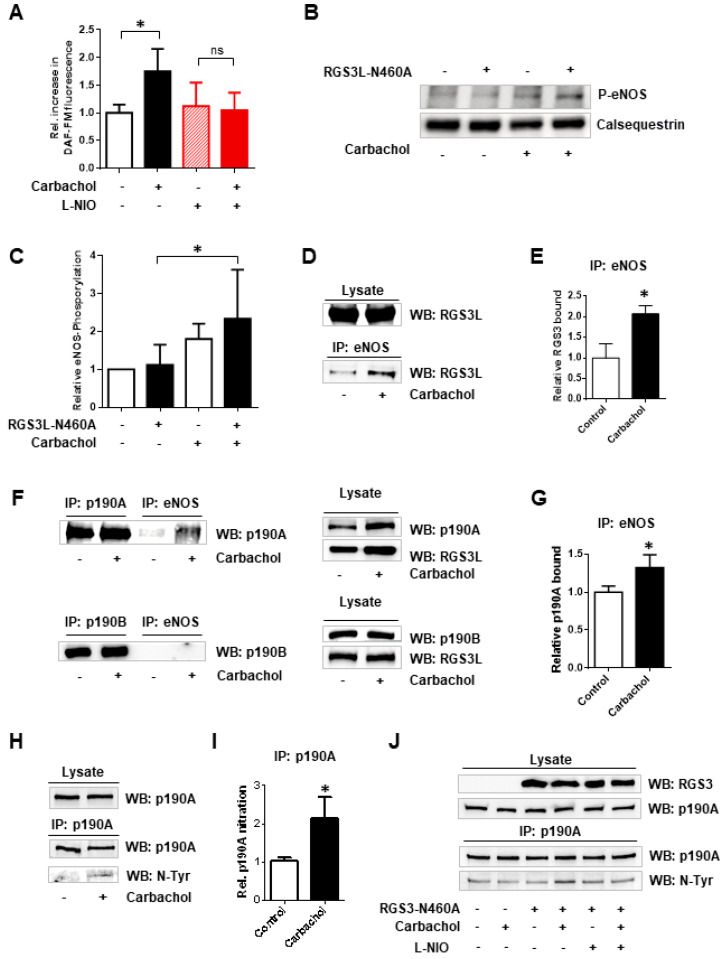
Carbachol-induced nitration of p190A by eNOS in NRCM. (**A**) Quantification of NO production in NRCMs by DAF-FM fluorescence. Cells were transduced with Ad-RGS3L-N460A for 48 h and additionally treated with 10 µM L-NIO or solvent for 15 min. Thereafter, the cells were stimulated with 1 mM carbachol or solvent for 5 min at 37 °C. Values are mean + SD (*n* = 3). * *p* < 0.05; one-way ANOVA with Tukey’s multiple comparison was performed. (**B**,**C**) Activating phosphorylation of eNOS. Cells were transduced with Ad-EGFP or Ad-RGS3L-N460A for 48 h. Thereafter, the cells were stimulated with 1 mM carbachol or solvent for 1 min at 37 °C and the eNOS phosphorylation was analyzed by immunoblotting using a phosphosite (Ser1177)-specific antibody. A representative experiment (**B**) and quantification of the relative amount of phosphorylated eNOS (P-eNOS) compared to the non-stimulated control (**C**) of *n* = 8 experiments are shown. Calsequestrin served as a loading control. Values are mean ± SD, * *p* < 0.05 (**D**–**G**) Binding of RGS3L-N460A and p190A to eNOS after carbachol stimulation. For the immunoprecipitation experiments, cells were transduced with Ad-RGS3L-N460A for 48 h and thereafter stimulated without and with carbachol for 5 min. The immunoprecipitation was performed with an anti-eNOS antibody. The total amounts of RGS3L-N460A, p190A, and p190B in the cell lysates served as loading controls. Representative experiments (**D**,**F**) and quantification of the relative amount of bound proteins compared to the non-stimulated control (**E**,**G**) are shown of *n* = 3 (**E**), *n* = 4 (**G**) experiments. Values are mean ± SD, * *p* < 0.05; unpaired Student’s *t*-test was performed. (**H**,**I**) Nitration of p190A after carbachol stimulation. Immunoprecipitation from total cell lysates of NRCM expressing RGS3L-N460A was performed with the anti-p190A antibody. Nitration of precipitated proteins was detected with an anti-nitro-tyrosine antibody. A representative experiment (**H**) and quantification of the relative amount of nitrated p190A proteins compared to the non-stimulated control (**I**) of *n* = 6 experiments are shown. Values are mean ± SD, * *p* < 0.05; unpaired Student’s *t*-test was performed. (**J**) Inhibition of the nitration of p190A by the eNOS inhibitor L-NIO. The experiment was performed as described before. For eNOS inhibition, cells were treated with 10 µM L-NIO or solvent for 15 min prior to the stimulation with carbachol.

Modulation of eNOS function is considered to be a promising pharmacological strategy in cardiovascular diseases [21,22]. It is known that the activation of eNOS can be controlled by cholinergic signaling mediated by the M_2_R in cardiac myocytes. For example, Feron et al. reported the dynamic targeting of the agonist-stimulated M_2_R to caveolae in cardiac myocytes, which leads to eNOS activation [7,8,11,12]. We therefore verified that carbachol-induced NO-production did occur in the NRCM under the conditions used herein to study RhoA activation. Using the NO-sensitive fluorescent dye DAF-FM, we observed an approximately two-fold increase in basal fluorescence upon stimulation of NRCM with carbachol. The carbachol-induced increase in DAF-FM fluorescence was completely suppressed by L-NIO treatment (Figure 3A). The activation of eNOS is regulated by the phosphorylation at Ser1177, which is targeted by several kinases, the phosphorylation of which can be monitored using phosphosite-specific antibodies [23,24]. In NRCM, we detected the transient carbachol-induced phosphorylation of eNOS at Ser1177, which was strongest after 1 min of stimulation and reached statistical significance in the presence of RGS3L (Figure 3B,C and Figure S2). As the data were in line with the reported M_2_R-induced eNOS activation, we next analyzed the complex formation of eNOS with p190A by co-immunoprecipitation. All antibodies used for the precipitation showed specificity against their target protein. Co-immunoprecipitation, e.g., eNOS with Cav3 [25], was verified against an unspecific IgG control (Figure S1). As shown in Figure 3D,E, in NRCM expressing high levels of RGS3L, the immunoprecipitation of eNOS resulted in the co-immunoprecipitation of RGS3L. Upon stimulation with carbachol, an approximately two-fold increase in the binding of RGS3L to eNOS was detected.

Interestingly, the immunoprecipitation of eNOS resulted also in the co-immunoprecipitation of p190A but not its paralog p190B (Figure 3F). Stimulation with carbachol increased the co-immunoprecipitation of p190A approximately 1.3-fold (Figure 3G). Within the same samples, the nitration of p190A was detected using an anti-nitrotyrosine-specific antibody. In line with the increased eNOS activation and enhanced binding of p190A to eNOS, a carbachol-induced increase in p190A nitration (> two-fold) was observed when RGS3L-N460A was overexpressed (Figure 3H–J). In accordance with the previously published data [16], this nitration was inhibited by treatment with L-NIO (Figure 3J). Taken together, these data indicate that, by stimulation of M_2_R in cardiac myocytes expressing high levels of RGS3L, the complex formation of eNOS with p190A and RGS3L is induced. The eNOS activation within this complex increases NO production, subsequent peroxynitrite formation [16], and thus the nitration of p190A. The sensitivity of the carbachol-incuced RhoA activation to the inhibition of eNOS suggests that the nitration reduces the GAP activity of p190A towards RhoA, as observed before in endothelial cells [16]. Based on our current data, we can, however, not exclude that the eNOS-induced formation of nitrogen or reactive oxygen species gives rise to other posttranslational modifications of the proteins involved in the signaling pathway studied herein.

### 3.2. The RGS3L-Dependent RhoA Activation after Carbachol Stimulation in NRCM Likely Occurs at Caveolae

**Figure 4:**
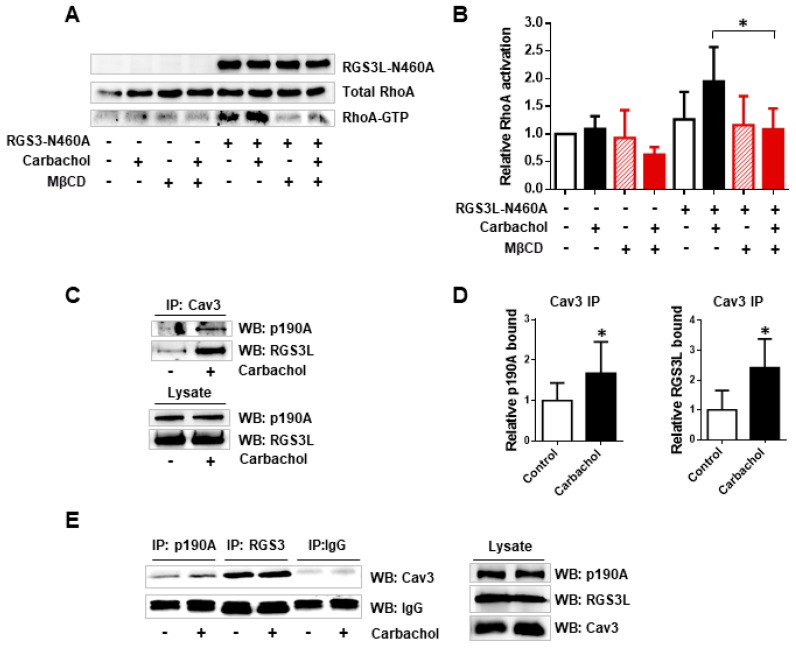
The role of caveolae and Cav3 in the carbachol-induced RhoA activation and interaction with p190A and RGS3L. (**A**,**B**) Effect of MβCD treatment on the carbachol-induced RhoA activation in NRCM. Cells were transduced with Ad-RGS3L-N460A or Ad-EGFP for 48 h and subsequently treated with 1 mM MβCD for 30 min prior to the stimulation with 1mM carbachol. The level of RhoA-GTP was measured by the RhoA activation assay. A representative experiment (**A**) as well as quantification of RhoA activity (RhoA-GTP/total RhoA) (**B**) are shown. The mean of RhoA-GTP levels detected in unstimulated cells was set to 1.0. Values are mean ± SD, *n* = 7. * *p* < 0.05; one-way ANOVA with Tukey’s multiple comparison was performed. Visualization (**C**) and quantification (**D**) of the Cav3–p190A and Cav3–RGS3L interaction using immunoprecipitation. Cells were transduced with Ad-RGS3L-N460A for 48 h and thereafter stimulated without and with 1 mM carbachol for 5 min. Immunoprecipitation was performed with the anti-Cav3 antibody. Bound p190A and RGS3L were detected with anti-p190A and anti-RGS3 antibody, respectively. The means of p190A and RGS3L levels detected in unstimulated cells were set to 1.0. Values are mean ± SD, *n* = 8/6. * *p* < 0.05; unpaired Student’s *t*-test was performed. (**E**) Reciprocal immunoprecipitation using anti-p190A and anti-RGS3 antibodies. Bound Cav3 was detected with the anti-Cav3 antibody. An unspecific IgG was used as a negative control. IgGs were visualized by immunoblotting in the precipitates.

It is known that M_2_Rs translocate into caveolae upon agonist binding [11,12]. Thus, we studied whether lipid rafts and caveolins play a role in the RGS3L-dependent RhoA activation after M_2_R stimulation. MβCD is a cholesterol-removing agent often used in cells, including NRCM [26], for lipid raft disruption. As shown in Figure 4A,B, MβCD treatment completely abolished the carbachol-induced RhoA activation in NRCM, especially in cells transduced with the adenovirus encoding RGS3L-N460A compared to the cells transduced with the control virus.

Cav3, one of the main structural proteins in caveolae in cardiac myocytes, including NRCM, is known to bind to various signal transducing molecules in caveolae [9]. To test for the possible complex formation of Cav3 with p190A and RGS3L, immunoprecipitation was performed in the lysates of NRCM transduced with the RGS3L-N460A-encoding adenovirus (Figure 4C,D). Both p190A and RGS3L were co-precipitated from NRCM lysates by a Cav3-specific antibody. Stimulation with carbachol significantly increased both the Cav3/p190A and the Cav3/RGS3L interaction 1.7- and 2.4-fold, respectively. Reciprocal immunoprecipation using anti-p190A and anti-RGS3 antibodies confirmed the co-immunoprecipation of Cav3 with p190A and RGS3L (Figure 4E).

**Figure 5:**
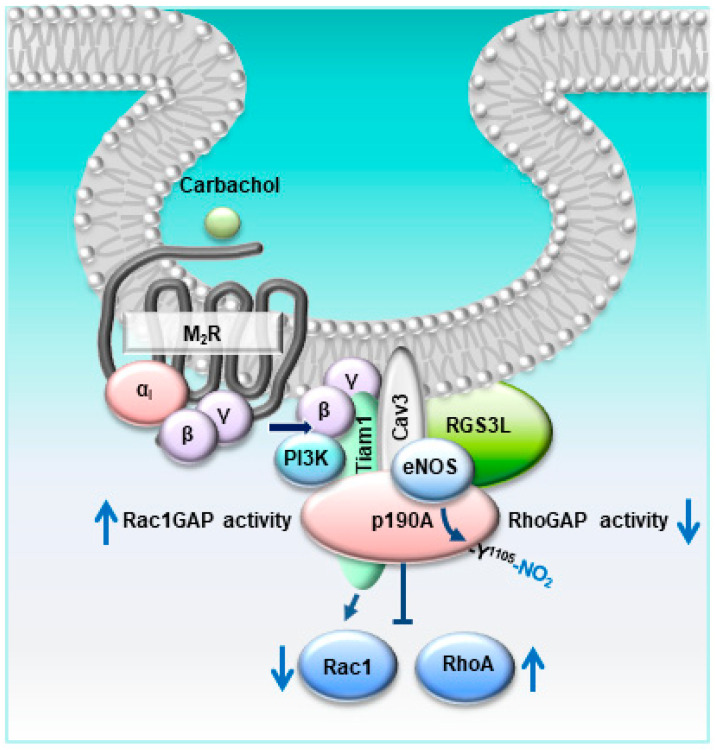
Schematic overview of the carbachol-induced nitration of p190A by eNOS in caveolae involved in the switch from Rac1 to RhoA activation in cardiac myocytes.

Taking our previously reported data into account, in which RGS3L/p190RhoGAP-dependent RhoA activation upon stimulation of M_2_Rs was established [13,17,19,20], the data reported herein point to a rather complex molecular mechanism by which M_2_Rs regulate Rac1 and RhoA activity in cardiac myocytes depending on the expression level of the RGS3 isoform RGS3L (Figure 5). At a low expression level of RGS3L, stimulation of M_2_Rs induces the activation of Rac1 via the canonical G_i_βγ- and phosphoinositide-3-kinase (PI3K)-mediated stimulation of the Rac1-specific guanine nucleotide exchange factor Tiam1. Under these conditions, p190A is primarily inactivating RhoA. At high expression levels of RGS3L, the concomitantly occurring M_2_R-induced activation of eNOS in caveolae [8,12] allows for peroxynitrite formation and the nitration of Y1105 in p190A [16]. Nitrated p190A binds to RGS3L and thus is found in complex with Cav3 and RGS3L. In accordance with the data observed in endothelial cells [16], the GAP activity of p190A towards RhoA is reduced, leading to enhanced RhoA activity after stimulation with carbachol. The concomitant increase in RacGAP activity (see Figure 1), the translocation in proximity to the Rac1 activator Tiam1, and the scavenging of activating G_i_βγ by RGS3L [13,17,27] most likely contribute to the strong suppression of carbachol-induced Rac1 activity. In accordance with this proposed mechanism, the carbachol-induced Rac1 activity in NRCM was found to be sensitive to pertussis toxin treatment, Gβγ scavenging, PI3K inhibition, as well as Tiam1 depletion [13,17]. Similarly, in accordance with this model, the carbachol-induced RhoA activation in NRCM was sensitive to the manipulations suppressing carbachol-induced Rac1 activation, but required high expression of RGS3L [13,17] and, as demonstrated herein, requires intact lipid rafts and carbachol-induced NO formation by eNOS associated with Cav3.

We reported recently that the increased RhoA activity in cardiac myocytes expressing high levels of RGS3L induced a positive inotropic response upon carbachol stimulation in ventricular strips of explanted rat hearts [13]. In line with these data, a positive inotropic response upon M_2_R stimulation was observed in the neonatal rat heart [20] or in the adult rat heart developing heart failure after myocardial infarction [28]. Whereas as a relatively high endogenous expression of RGS3L was evident in the neonatal rat heart, an increase in its expression was observed in failing compared to non-failing hearts [29]. Based on the data shown herein, also NO production by eNOS likely contributes to the increase in RhoA activity and thus potentially to increased contractility. However, the modulatory effects of NO on contractile function appear to be complex and controversial [3]. On the other hand, studies in eNOS^−/−^ mice reported that eNOS is not necessary for basal cardiac contractility, but β-adrenergic contractility was increased in eNOS-deficient hearts [30,31]. It was also found that eNOS is unimportant for the anti-adrenergic effect of ACh and adenosine, whereas others reported that eNOS plays a role in the regulation of the myocyte L-type voltage-sensitive calcium channel current (I_Ca-L_) by muscarinic receptors in cardiac myocytes [32]. Moreover, cardiac NO production was reported to be activated by stretch and to enhance intracellular Ca^2+^ transients, causing an increase in contraction in response to stretch [3]. A functional interaction between the myocardial NOS isoforms was also discussed [33]. Based on these reports describing multiple and divergent functional effects of NO in the heart and its effects on the vasculature, it does not seem feasible to envision an unspecific exogenous NO supply to enforce the herein described NO-/peroxynitrite-mediated RhoA activation as an inotropy-enhancing pathway and thus a future therapeutic option [13]. Nevertheless, a novel nitric oxide donor, S-Nitroso-N-Pivaloyl-D-Penicillamine (SNPiP), was reported to increase cardiac performance in mice [34]. As an underlying mechanism, the induction of the non-neuronal cardiac cholinergic system (NNCCS), which allows the production and secretion of ACh directly from the cardiac myocytes, was identified. The NNCCS is involved in the maintenance of cardiac function and was reported to protect against oxidative stress, hypertrophy, and heart failure [35]. As the ventricles in the heart are lacking parasympathetic innervation, the M_2_Rs expressed in ventricular cardiac myocytes are likely activated by ACh provided by the NNCCS. Therefore, our new mechanism describing eNOS-dependent RhoA activation in the presence of RGS3L by muscarinic receptor stimulation in cardiac myocytes might be one facet of the effects of SNPiP application on cardiac performance.

## 4. Conclusions, Study Limitations, and Further Perspectives

Based on our previous work showing that M_2_Rs can induce RhoA activation dependent on the expression of RGS3L and p190RhoGAP [13,17,20], we intended to clarify the mechanism by which these Gi/o-coupled receptors regulate the activity of the GAP to switch its substrate specificity from RhoA to Rac1. The molecular mechanism depicted in Figure 5 explains how altering the RGS3L expression in cardiac myocytes manipulates RhoA/Rac1 activity. The herein detected involvement of caveolar eNOS as a required mediator in the nitration of p190ARhoGAP offers the additional possibility to interfere with this pathway with NO donors and NOS inhibitors. The functional consequences, when the carbachol-induced Rac1 activity is decreased whereas the RhoA activity is increased, have been described on the cellular level in [13]. The production of reactive oxygen species and hypertrophic responses are decreased, whereas the activated RhoA/Rho-dependent kinase (ROCK) pathway allows for an increase in contractility [13]. ROCK-mediated inotropy upon carbachol stimulation was observed in neonatal rat hearts [20] and a rat heart failure model after myocardial infraction [28], indicating that this pathway might be of physiological relevance in the developing heart and might be reactivated in the diseased heart.

Nevertheless, all these data have clear limitations in their significance. We cannot predict whether RGS3L-N460A expression and the modulation of p190ARhoGAP nitration can be developed into novel interventions for the treatment of heart failure. This has to be tested, for example, in mouse or rat heart failure models and analyzed in vivo, e.g., by echocardiography, to assess its influence on cardiac contractility. In addition, the likely co-occurring beneficial effect of the inhibition of Rac1-dependent signaling can also be analyzed in such models in more detail. We are pursuing such a strategy in our laboratory.

## Data Availability

The datasets generated during and/or analyzed during the current study are available from the corresponding author on reasonable request.

## Supplementary Materials

The following supporting information can be downloaded at: https://www.mdpi.com/article/10.3390/cells12202432/s1, Figure S1: Antibody specificity for immunprecipitation; Figure S2: Time course of eNOS phosphorylation in NRCM.
