# Supplementary material for: The Muscarinic Acetylcholine M2 Receptor-Induced Nitration of p190A by eNOS Increases RhoA Activity in Cardiac Myocytes
Source: Cells. 2023 Oct 11;12(20):2432. doi: 10.3390/cells12202432 (PMC10605742; doi:10.3390/cells12202432)
Supplement: Supplementary file 1 [file cells-12-02432-s001.zip › cells-2623923-supplementary.pdf]

Figure S1: Antibody specificity for immunoprecipitaion

Cav3 CoIP negative control experiment and verification of eNOS-Cav3 interaction

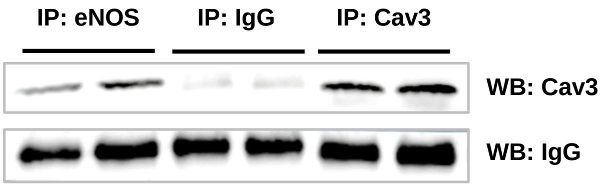

RGS3 negative control experiment

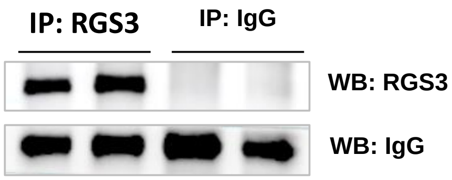

p190A negative control experiment

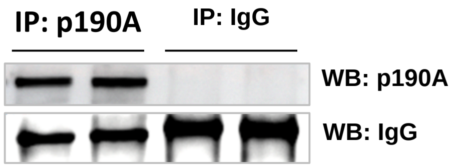

eNOS negativ control experiment

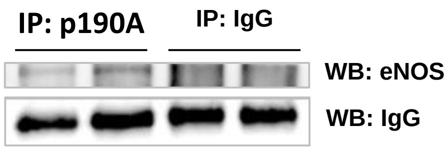

Cells were transduced with Ad-RGS3L-N460A for 48 h. Immunoprecipitation were performed using anti-Cav3, anti-RGS3 and anti-p190A antibodies. Precipitated and co-precipitated proteins were detected with the respective antibody. An unspecific IgG was used as negative control. IgGs were visualized by immunoblotting in the precipitates.

Figure S2: Time course of eNOS phosphylation in NRCM

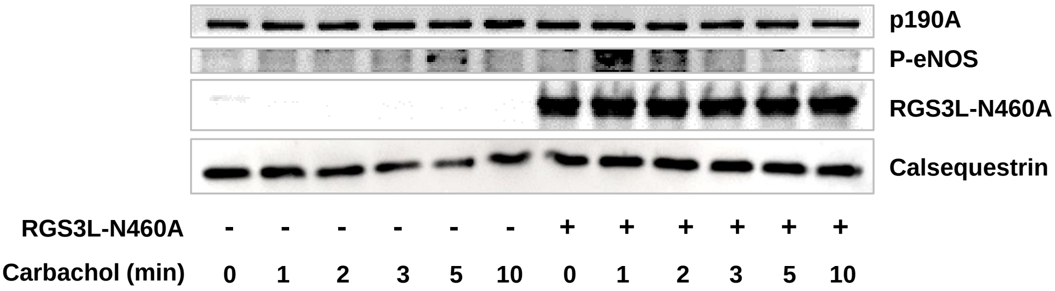

Cells were transduced with Ad-EGFP or Ad-RGS3L-N460A for 48 h. Thereafter, the cells were stimulated with 1 mM carbachol or solvent for the indicated periods of time at 37°C. The eNOS phosphorylation was analyzed by immunoblotting using a phosphosite (Ser1177) specific antibody. Calsequestrin and p190A served as loading controls.
